# Cesarean Section

**Published:** 1916-06

**Authors:** 


**Cesarean Section.** In 1879, Felkin, an African traveler, witnessed a cesarean section performed by the natives in the heart of Uganda. The woman was held in a reclining posture by two men. At her side was a gourd of banana wine, and she was half drunk. The operator stood at her left. First he washed his hands in banana wine, then he washed the belly with the same—active antiseptic measures. With a short curved knife he made one incision thru the belly, right into the uterus and quickly delivered the child alive, an assitant holding the uterine incision open by hooking his fingers into it. By uterine massage, the placenta was expressed and hemorrhage controlled. Several bleeding points were cauterized with a hot iron. The cervix was dilated from above with the fingers. The assistants then turned the patient on her side to allow the blood to drain out of the peritoneal cavity, the intestines being retained by a square of plaited twigs, after which the belly was sewed up with pins and figure-of-eight sutures. The pins were made from bamboo stick, the sutures from reed fibres. The wound was covered with a paste made of aromatic herbs. The patient recovered in eleven days, having run a mild febrile course. Without doubt, this operation must have been performed for many centuries for the technique to be so perfectly developed. —DeLee, Illinois Medical Journal.

